# Identification of key candidate targets and pathways for the targeted treatment of leukemia stem cells of chronic myelogenous leukemia using bioinformatics analysis

**DOI:** 10.1002/mgg3.851

**Published:** 2019-08-02

**Authors:** Huayao Li, Lijuan Liu, Jing Zhuang, Cun Liu, Chao Zhou, Jing Yang, Chundi Gao, Gongxi Liu, Changgang Sun

**Affiliations:** ^1^ College of Basic medical Shandong University of Traditional Chinese Medicine Jinan Shandong PR China; ^2^ College of First Clinical Medicine Shandong University of Traditional Chinese Medicine Jinan Shandong PR China; ^3^ Department of Oncology Affilited Hospital of Weifang Medical University Weifang Shandong PR China; ^4^ Department of Oncology Weifang Traditional Chinese Hospital Weifang Shandong PR China

**Keywords:** bioinformatics, chronic myeloid leukemia, differentially expressed genes, differentially expressed microRNAs, gene chip, leukemia stem cells

## Abstract

**Background:**

Chronic myelogenous leukemia (CML) is a myeloproliferative neoplasm that arises from the acquisition of constitutively active BCR‐ABL tyrosine kinase in hematopoietic stem cells. The persistence of bone marrow leukemia stem cells (LSCs) is the main cause of TKI resistance and CML relapse. Therefore, finding a key target or pathway to selectively target LSCs is of great significance for the thorough treatment of CML.

**Methods:**

In this study, we aimed to identify key microRNAs, microRNA targets and pathways for the treatment of CML LSCs by integrating analyses of three microarray data profiles. We identified 51 differentially expressed microRNAs through integrated analysis of GSE90773 and performed functional gene predictions for microRNAs. Then, GSE11889 and GSE11675 were integrated to obtain differentially expressed genes (DEGs), and the overlapping DEGs were used as models to identify predictive functional genes. Finally, we identified 116 predictive functional genes. Clustering and significant enrichment analysis of 116 genes was based on function and signaling pathways. Subsequently, a protein interaction network was constructed, and module analysis and topology analysis were performed on the network.

**Results:**

A total of 11 key candidate targets and 33 corresponding microRNAs were identified. The key pathways were mainly concentrated on the PI3K/AKT, Ras, JAK/STAT, FoxO and Notch signaling pathways. We also found that LSCs negatively regulated endogenous and exogenous apoptotic pathways to escape from apoptosis.

**Conclusion:**

We identified key candidate targets and pathways for CML LSCs through bioinformatics methods, which improves our understanding of the molecular mechanisms of CML LSCs. These candidate genes and pathways may be therapeutic targets for CML LSCs.

## 1 INTRODUCTION

Chronic myelogenous leukemia (CML) is a stem cell cancer that develops as a result of the *t*(9;22) translocation in hematopoietic stem cells (HSCs) (Nowell & Hungerford, 1961; Rowley, 1973). This translocation results in constitutive expression of the fusion tyrosine kinase BCR‐ABL1(Heisterkamp et al., 1983) and transformation of HSCs into leukemic stem cells (LSCs). The development of effective tyrosine kinase inhibitors (TKIs), such as imatinib, was a milestone in targeted therapy for CML (O'Brien et al., 2003). However, recent evidence suggests that in approximately 50%–60% of lifelong TKI patients, LSCs persist (minimal residual disease is maintained by a subpopulation of LSC in the bone marrow); this is a primary cause of TKI resistance (Chomel et al., 2011; Chu et al., 2011; Holyoake, Jiang, Eaves, & Eaves, 1999), and if TKI treatment is discontinued, it can serve as a reservoir for disease recurrence (Chen & Kang, 2015; Copland et al., 2006; Jorgensen, Allan, Jordanides, Mountford, & Holyoake, 2007). Therefore, to improve the cure rate of CML, there is a significant need to develop new therapeutic methods that target LSCs.

MicroRNAs are small noncoding RNAs that participate in the posttranscriptional regulation of gene expression. MicroRNAs function as negative regulators by binding to the 3′‐untranslated region of candidate mRNAs and repress gene expression by inhibiting protein translation or degrading mRNAs (Chen & Kang, 2015). Gene chip technology is a rapidly developing technology and combines molecular biology, genetics, nucleic acid chemistry, computer software and micromechanical automation technology, making the discipline a highly integrated product of many fields (Venkataraman, Vasudevan, & Gupta, 2014). The use of gene chip technology to study CML involves a variety of genetic variants, and gene chip technology can compare expression changes of HSC microRNAs in normal HSCs and chronic myeloid leukemia patients, allowing the identification of key microRNAs in the development and progression of CML. New biomarkers for CML and candidate therapeutic targets provide new ideas for the clinical treatment of CML (Ushijima et al., 2013). With the widespread use of gene chips, most of the slice data on CML have been stored on public data platforms. For example, researchers collected genetic microarray data of CML at various times and concluded that *MLLT4*, *WDR35*, *EPHB4*, integrin‐mediated cell adhesion, focal adhesion and the regulation of the actin cytoskeleton are principal genes and pathways during CML progression (Zhang, Liu, Lin, Pan, & Xu, 2014). However, in independent studies, the results were limited or inconsistent due to the heterogeneity of the tissues or samples. Nevertheless, combining bioinformatics methods with expression profiling techniques can innovatively address these shortcomings. For example, researchers integrated four cohort profile datasets to elucidate the potential key candidate genes and pathways in colorectal cancer (Guo, Bao, Ma, & Yang, 2017). Another integrated cohort profile dataset identified potential crucial genes and pathways associated with the carcinogenesis of renal cell carcinoma and further analyzed the molecular mechanisms implicated in its tumorigenesis (Song et al., 2018).

To further elucidate the molecular mechanisms of LSC carcinogenesis in CML and to screen CML biomarkers and key candidate targets, we screened differentially expressed microRNAs using microarray data and predicted the target of microRNAs through the miRTarBase database and TargetScanHuman database (http://www.targetscan.org/vert_71/). Subsequently, to increase the accuracy of the prediction target, gene expression on two gene chips was compared based on the gene microarray data, common differentially expressed genes (DEGs) were screened, DEGs and predicted targets were intersected, and the microRNA targets were screened for the next study. The three gene chips were from National Center of Biotechnology Information (NCBI) gene expression omnibus (GEO) database (https://www.ncbi.nlm.nih.gov/geo). The three original microarray datasets included the expression profiles GSE90773 (Salati et al., 2017), GSE11889 (Bruns et al., 2009), GSE11675 (Lemoli et al., 2009), with 20 cases of CML patients. On a total of 36 matched CML patients with bone marrow LSCs and normal bone marrow HSCs, analysis was carried out using GEO2R, an online analysis tool for the GEO database that is based on R language. Through analysis and integration identification, we obtained predict targets of differentially expressed microRNA for further study. Subsequently, we used gene ontology and pathway enrichment analyses to screen DEGs using Cluego, a plug‐in of Cytoscape software, as well as websites such as QuickGo (https://www.ebi.ac.uk/QuickGO/), Gene Ontology (http://www.geneontology.org/), Kegg Pathway (http://www.genome.jp/kegg), WikiPathways (https://www.wikipathways.org/), Reactome ( https://reactome.org/); these analyses were employed to assist with Go analysis and KEGG analysis. Strings (https://string-db.org) were used to construct the DEG protein interaction network. CytoNCA, an analysis plug‐in for Cytoscape 3.5.1, was used to topologically analyze the DEGs; MCODE, an analysis tool for Cytoscape 3.5.1, was employed to perform module analysis of the protein network. Via protein network construction, topology analysis and module analysis integration were employed to identify hub genes in CML. It is expected that in this study, the biomarkers and pathways identified in CML may reveal the potential molecular mechanisms of LSC canceration in CML. The identified new key candidate targets provide new ideas and methods for the treatment of CML.

## 2 MATERIALS AND METHODS

### 2.1 Gene chip microarray data and differentially expressed microRNA identification

The Gene Expression Omnibus (GEO, http://www.ncbi.nlm.nih.gov/geo/) is an international public repository for high‐throughput microarray and next‐generation sequence functional genomic data sets submitted by the research community. GEO2R is an R‐based web application which can analyze GEO data (Barrett et al., 2013). We downloaded CML bone marrow LSCs and normal bone marrow HSC gene expression profiles of GSE90773 from the GEO database. GSE90773 data are based on the GPL19066 (Exiqon microRNA Ready‐to‐Use PCR, Human panel I+II, V3.R) platform, which contains 10 CML patient marrow LSCs and eight normal bone marrow HSCs (Submission date 1 Dec 2016).

We used the GEO2R online analysis tool of the GEO database to identify statistically significant differentially expressed microRNAs between CML patient marrow LSCs and normal bone marrow HSCs. Statistically significantly differentially expressed microRNAs were defined using *p* < .05 and [logFC] > 2 as the cut‐off criteria. Employing HemI 1.0.3.7‐Heatmap Illustrator, we developed a heat map of differentially expressed microRNAs.

### 2.2 Gene chip microarray data and DEGs identification

We downloaded CML bone marrow LSCs and normal bone marrow HSCs gene expression profiles of GSE11889 and GSE11675 from the GEO database. GSE11889 data is based on the GPL571 ([HG‐U133A_2] Affymetrix Human Genome U133A 2.0 Array) platform, including seven CML patient marrow LSCs and five normal bone marrow HSCs (Submission date Jun 26, 2008). GSE11675 data is based on the GPL8300 ([HG_U95Av2] Affymetrix Human Genome U95 Version 2 Array) platform, including three CML patient marrow LSCs and three normal bone marrow HSCs (Submission date 4 Jun 2008). We selected these two microarray datasets for comprehensive analysis; both groups were based on gene expression profiles of HSC in the bone marrow. We used the GEO2R online analysis tool identify statistically significant DEGs between CML patient marrow LSCs and normal bone marrow HSCs. Statistically significant DEGs were defined as *p* < .05 and [logFC] > 1 as the cut‐off criteria.

Venny 2.0.2 (http://bioinfogp.cnb.csic.es/tools/venny/index2.0.2.html) is a free online website. To enhance the accuracy of the research results, we screened out common DEGs of two high‐throughput microarray databases using Venny 2.0.2.

### 2.3 Identification the predict target genes of differential expression microRNAs

We employed two databases, miRTarBase (http://mirtarbase.mbc.nctu.edu.tw/index.html) (Chou et al., 2016), and TargetScanHuman database (http://www.targetscan.org/vert_71/) (Qin et al., 2017), to predict targets for differentially expressed microRNAs. We identified overlapped target genes to further increase the reliability of the bioinformatics analysis. Using Venny 2.0.2, we intersected the predicted target and identified DEGs, thus increasing the confidence of the predicted target genes. We used Cytoscape software to build a microRNA‐target visual network.

### 2.4 Gene ontology and pathway enrichment analysis of targets of differential expression microRNAs

To further understand the related functions of the target genes of differentially expressed microRNAs to illustrate the pathogenesis of CML, we performed gene ontology and pathway enrichment analyses of the target genes. ClueGO, a plug‐in of Cytoscape software, is updatable with the newest files from Gene Ontology, KEGG, WikiPathways and Reactome. We performed Go analysis and KEGG analysis of the targets based on the Cluego plug‐in. When we ran the ClueGO plug‐in and selected the cut‐off threshold of the display path as *p* < .05.

### 2.5 Construction of protein interaction networks and identification of key candidate targets

To further elucidate the interactions between the microRNA target genes, we constructed a protein interaction network. Subsequently, the network was topologically analyzed to identify key candidate target genes, and pathway enrichment analysis was performed on key candidate target genes to elucidate key pathways that were activated during the pathogenesis of CML. We used the String database (https://string-db.org/cgi/input.pl) and Cytoscape software to build target protein networks and analyze the network (Su, Morris, Demchak, & Bader, 2014). We employed CytoNCA and the MCODE plug‐in to perform topological analysis and module analyses of the target network, respectively. The two analyses were used in combination to identify major nodes. The CytoNCA plug‐in performs topology analysis based on “betweenness (BC)”, “closeness(CC)”, “eigenvector(EC)”, “local average connectivity‐based method(LC)”, “network(NC)”, “subgraph(SC)”, “information(IC)” (ElHady, Abdel‐Halim, Abadi, & Engel, 2017). The plug‐in MCODE was used with the default parameters (degree cut‐off ≥ 2, node score cut‐off ≥ 0.2, K‐core ≥ 2, and max depth = 100) (Ashburner et al., 2000). We then found the corresponding microRNAs based on identified major targets to build a visualization network.

## 3 RESULTS

### 3.1 Identification of differentially expressed microRNAs in CML

**Figure 1:**
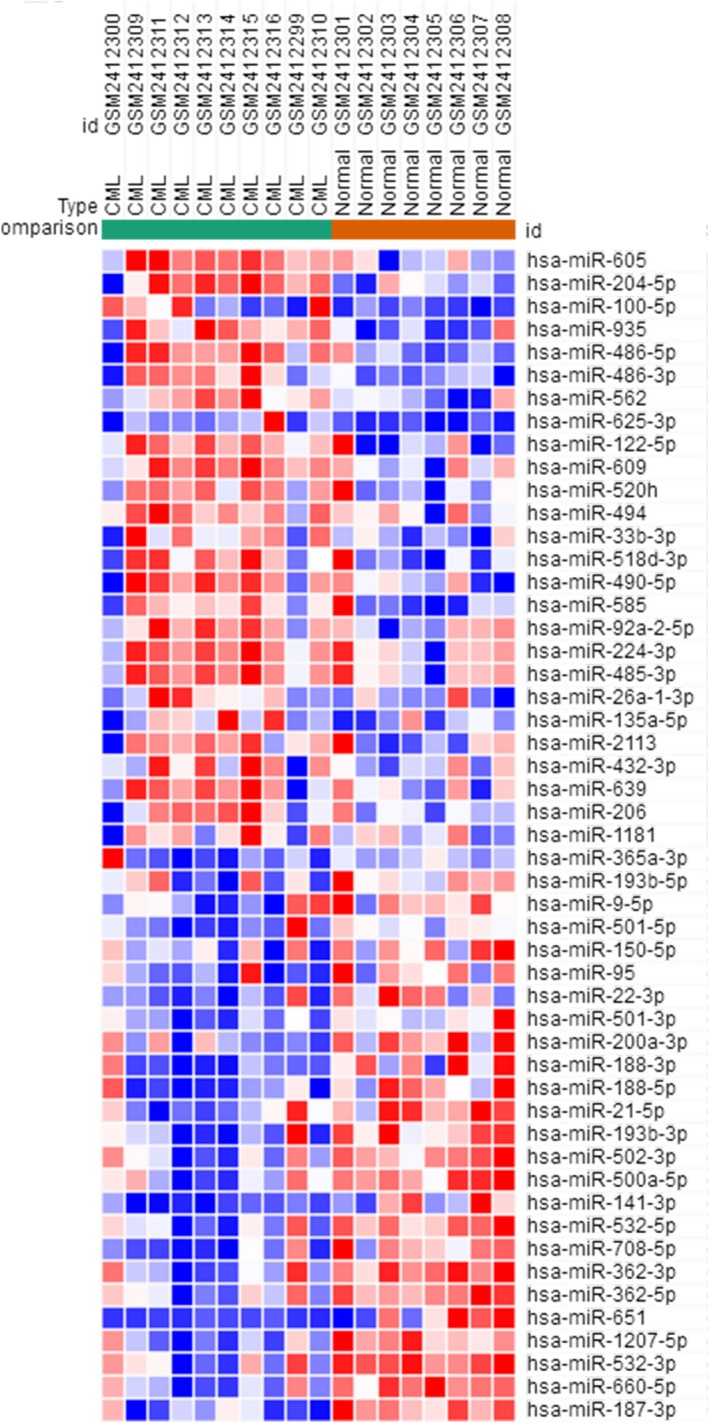
Heatmap of differentially expressed microRNAs of GSE90773 profile datasets

The GSE90773 data expression profile was downloaded from the NCBI‐GEO database, including bone marrow LSCs from 10 CML patients and bone marrow HSCs from eight normal patients. Microarray data were analyzed based on GEO‐GEO2R, and differentially expressed microRNAs were identified according to *p* < .05 and absolute logFC > 2 as cut‐off standards. A total of 51 differentially expressed microRNAs were identified, including 26 up‐regulated and 25 down‐regulated differentially expressed microRNAs. Subsequently, using Morpheus software, we developed 26 up‐regulated and 25 down‐regulated DEG heat maps (Figure 1).

### 3.2 Identification of DEGs in CML

**Figure 2:**
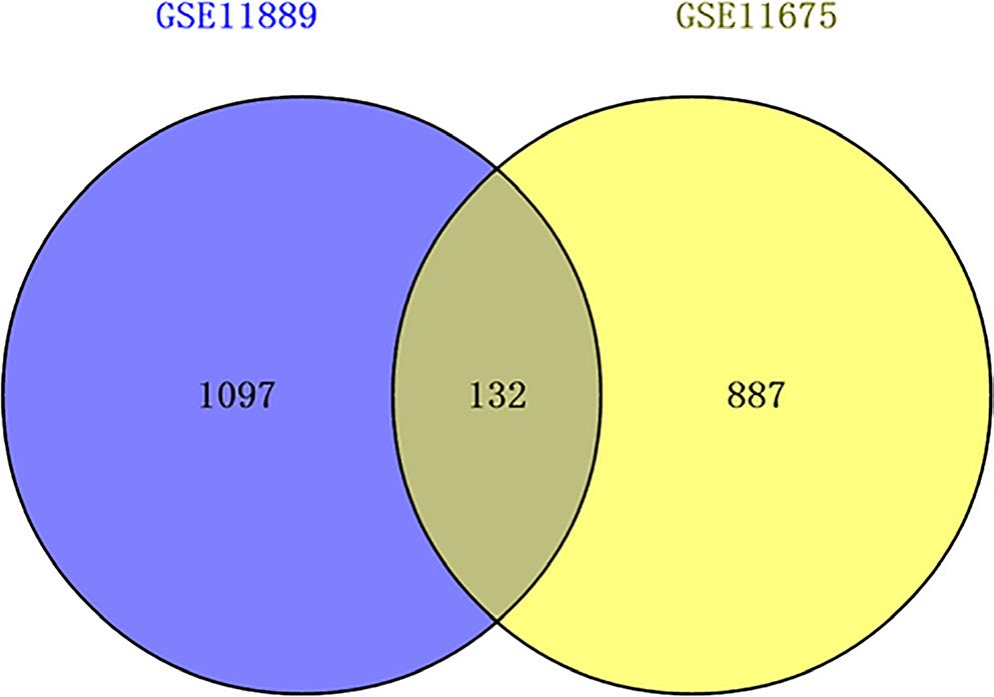
Overlapping 132 DEGs were identified from two microarray data profiles (GSE11889, GSE11675). Different color areas represented different microarray data profiles. The cross areas meant the commonly changed DEGs. DEGs, differentially expressed genes

GSE11889 and GSE11675 expression profiles were downloaded from the GEO database, including 10 CML patient marrow LSCs and eight normal bone marrow HSCs. We used the GEO2R online analysis tool identify statistically significant DEGs between CML patient marrow LSCs and normal bone marrow HSCs. Statistically significant DEGs were defined as *p* < .05 and [logFC] > 1 as the cut‐off criteria. Finally, 1,229 DEGs (containing 912 up‐regulated DEGs and 317 down‐regulated DEGs) were analyzed by analyzing the GSE11889 microarray data expression profile (Table S1); and 1,019 DEGs (containing 673 up‐regulated DEGs and 346 down‐regulatedDEGs) were obtained by analyzing the GSE11675 microarray data expression profile (Table S2). We screened out the common DEGs of the two high‐throughput microarray databases using Venny 2.0.2. We obtained a total of 132 DEGs that were common for gene the expression profiles GSE11889 and GSE11675 (Figure 2). A total of 132 DEGs were used as the identification criteria for the differentially expressed microRNA prediction targets.

### 3.3 Identification the predict target genes of differential expression micrornas

**Figure 3:**
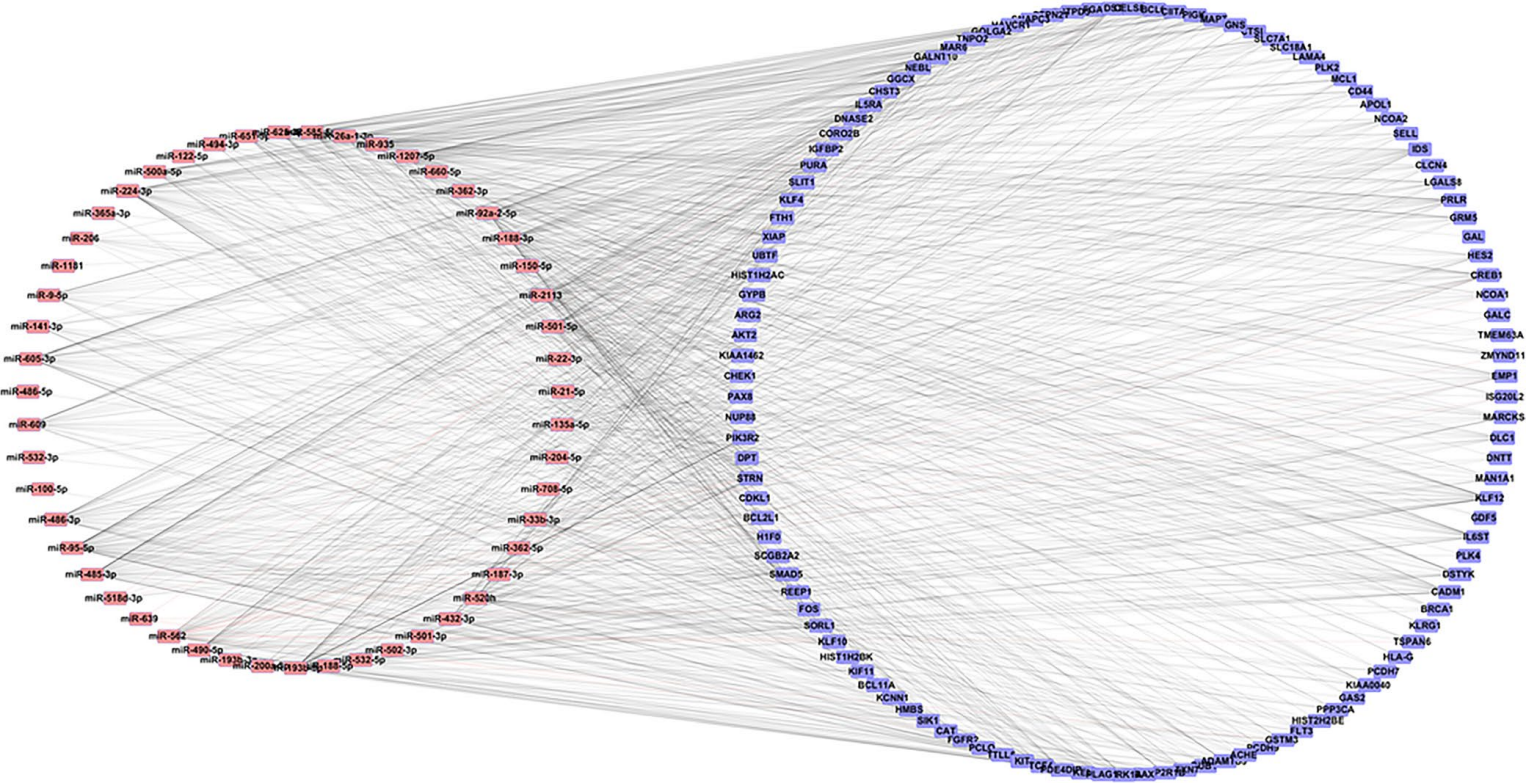
The microRNA‐Targets visualization network includes 51 differentially expressed microRNAs and 116 identified targets. The network contains a total of 167 nodes and 1,010 edges

We used miRarBase and the TargetScanHuman database to predict the target genes of differentially expressed microRNAs. Because of the complexity of the microRNA action, each microRNA can regulate many different types of genes. Therefore, we increased the confidence of the predict target genes by intersecting the predicted target with the identified DEG using Venny 2.0.2. Finally, we identified 116 microRNA target genes for further study. We defined the regulatory criteria for 116 target genes using the regulatory criteria of the GSE11889 microarray gene expression profile. There were 79 up‐regulated DEGs and 37 down‐regulated DEGs in the 116 targets. We used Cytoscape software to build a microRNA‐Target visual network (Figure 3). The microRNA‐Target network contained 168 nodes and 1,010 edges.

### 3.4 Gene ontology and pathway enrichment analysis of 116 target genes

To understand the features and functions of the 116 target genes, we employed the Cytoscape plug‐in ClueGo to perform Go analysis and pathway enrichment analysis of the target genes.

**Figure 4:**
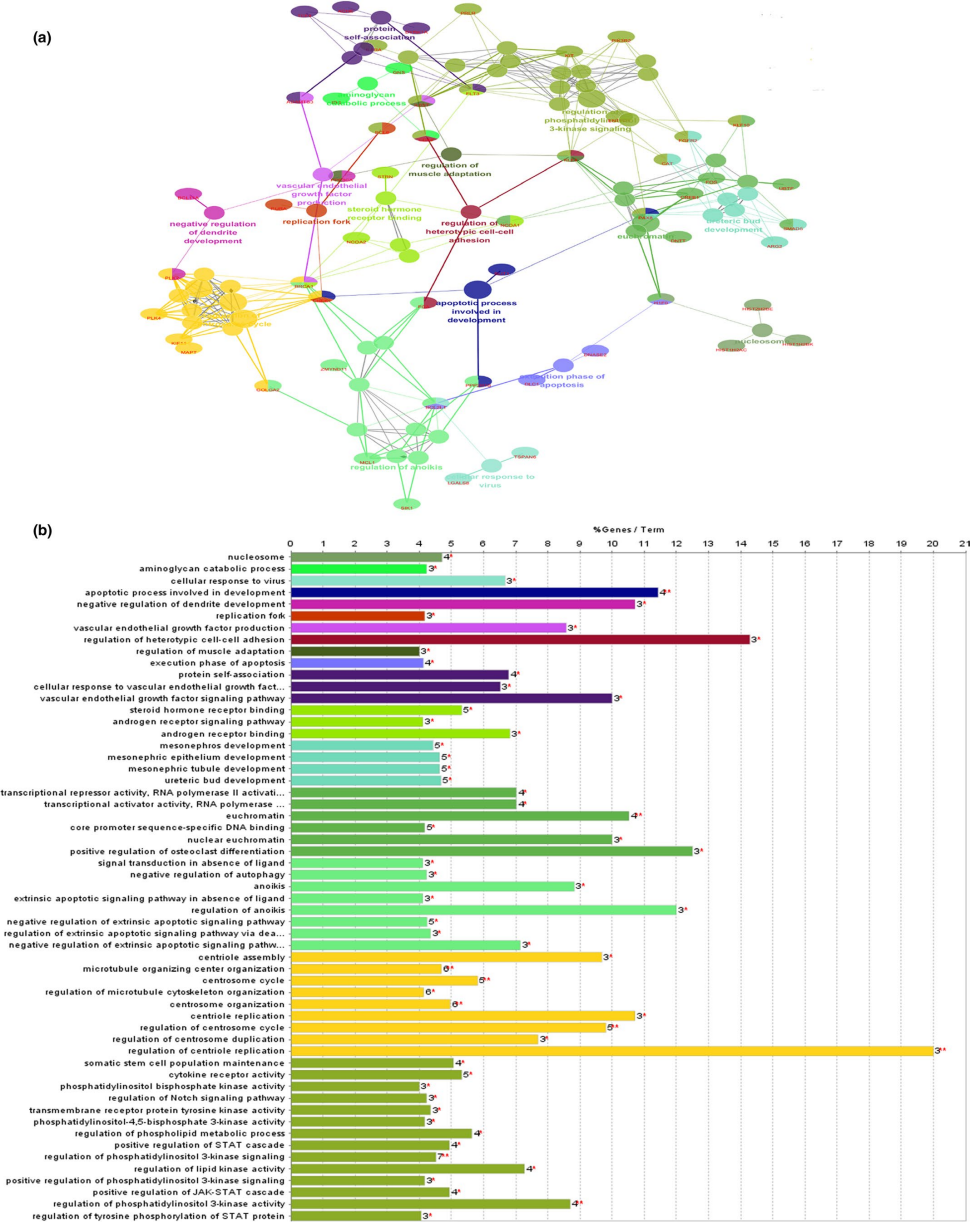
Gene ontology analyses of 116 identified targets in CML. We employed the Cytoscape plug‐in ClueGO for Gene ontology analysis with a truncation criterion of *p* < .05. (a) Gene ontology network; (b) Column annotation map of the Gene ontology network

Go analysis (including Molecular Function, Biological Process and Cellular Component) was performed on 116 target genes, with *p* < .05 as the cut‐off criterion (Ashburner et al., 2000; Su et al., 2014). After analysis, we performed Go analysis of 116 target genes (Figure 4), including BP, MF, and CC. A total of 57 Go analysis results were obtained. The analysis results were primarily focused on a single cellular biological process that was of molecular function. The enrichment results mainly focused on cell transcription, receptor binding activity and the activities of various enzymes, such as tyrosine kinase and phosphokinase. The biological processes involved the regulation of the amino acid and protein as well as regulation of cell proliferation. For a series of processes that regulate apoptosis, interestingly, there was dual regulation of endogenous apoptosis and extrinsic apoptosis. Surprisingly, in exogenous apoptosis, the signaling pathways were negatively regulated to achieve apoptosis escape, as those that may be associated with CML LSCs.

**Figure 5:**
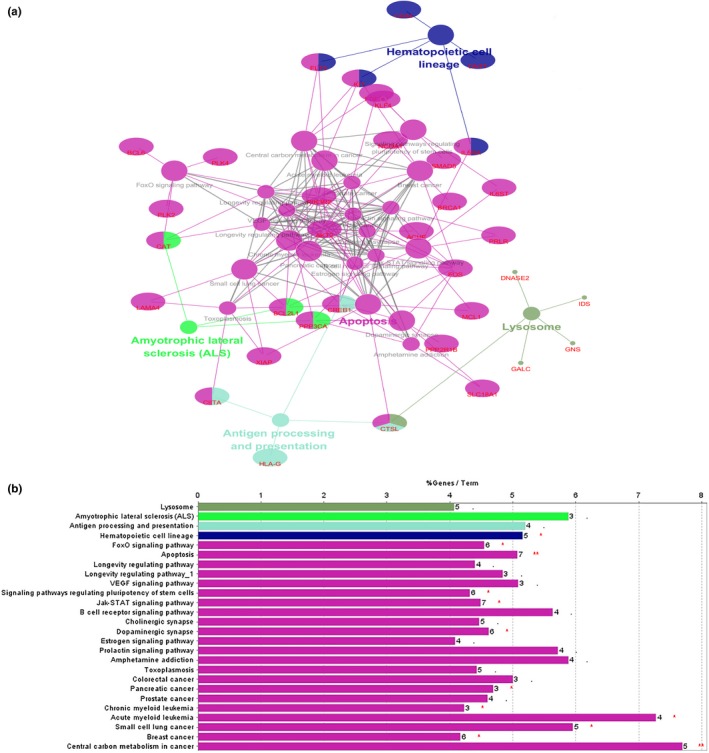
KEGG pathway analyses of 116 identified targets in CML. We employed the Cytoscape plug‐in ClueGO for gene ontology analysis with a truncation criterion of *p* < .05. (a) KEGG pathway network; (b) columnar annotation map of KEGG pathway network. CML, chronic myelogenous leukemia

KEGG pathway enrichment analysis of the 116 target genes was also performed in the ClueGo plug‐in, for which the cut‐off criterion was *p* < .05. We constructed an enrichment pathway network (Figure 5), obtaining a total of 26 enriched pathways. Pathway enrichment was mainly concentrated in signaling pathways that regulate the pluripotency of stem cells, hematopoietic cell lineages, chronic myeloid leukemia and acute myeloid leukemia. These pathways were closely related to the occurrence and development of leukemia. They also included some cancer‐related pathways, such as those for small cell lung cancer, breast cancer, and others. Also enriched were the FoxO signaling pathway, Jak‐STAT signaling pathway and apoptosis signaling pathway. The discovery of these pathways helps to further understand the mechanism of LSCs in the pathogenesis of CML.

### 3.5 Protein interaction networks and key candidate targets

**Figure 6:**
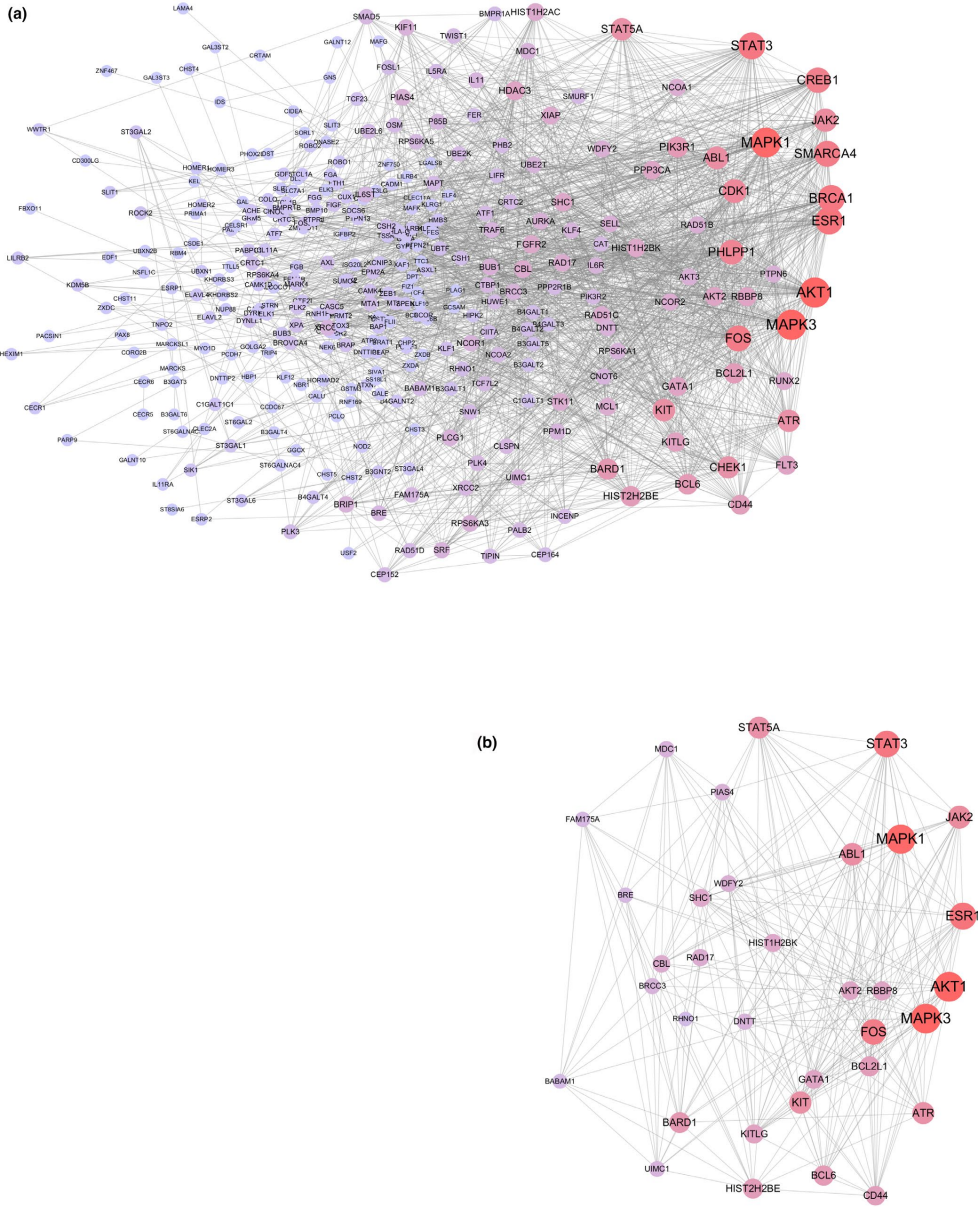
Protein interaction network construction and module analysis of identified genes, (a) The protein interaction network of 116 identified genes, the color depth of the gene deepens with the “degree” of the node, and the size of the node increases with the “degree”. (b) The most significant module in the network, including 31 nodes and 215 edges, the module function is closely related to the chronic myeloid leukemia and hematopoietic cell lineage

The construction of a protein interaction network is a way to quickly analyze the interactions between genes. We employ String online website (http://string-db.org) to build an interactive network of 116 targets. The restrictive condition is “human species”, the network is constructed and the “TSV” format file of the gene interaction relationship is downloaded, and then the file is imported into Cytoscape software for module analysis and topology analysis. We have obtained a protein network with 340 nodes and 1,839 edges (Figure 6a).

Using the Cytoscape plug‐in MCODE to analyze the network diagram module, the module selection criteria were as follows: degree cut‐off ≥ 2, node score cut‐off ≥ 0.2, K‐core ≥ 2, and max depth = 100. We chose one of the most significant modules for further analysis. This module had 31 nodes and 215 edges (Figure 6b). By conducting pathway enrichment analysis of the nodes, we found that these nodes were primarily enriched in pathways such as CML, acute myeloid leukemia and hematopoietic cell lineages. In addition, these nodes involved multiple cancer pathways specific to various signaling pathways, including the ErbB signaling pathway, Jak‐STAT signaling pathway, chemokine signaling pathway and T cell and B cell receptor signaling pathways. Interestingly, we found that eight genes in this module were derived from the 116 targets, including *DNTT*, *KIT*, *FOS*, *HIST1H2BK*, *HIST2H2BE*, *CD44*, *BCL6* and *BCL2L1*. These genes had higher “Degree” values in the module, which indicated that the gene had meaning (ElHady et al., 2017).

We used the Cytoscape's plug‐in CytoNCA to analyze the protein interaction network based on “betweenness (BC)”, “closeness(CC)”, “eigenvector(EC)”, “local average connectivity‐based method(LC)”, “network(NC)”, “subgraph (SC)”, and “information (IC)”. Standards were topologically analyzed and subsequently identified as 30 nodes, which were defined as major nodes. Among the 30 nodes (Table S3), 11 major nodes belonged to 116 targets, including *CREB1, KIT, MCL1, FOS, BRCA1, FLT3, CD44, AKT2, CHEK1, BCL6,* and *BCL2L1* (Table 1). Enrichment analysis of the 11 major genes was performed using the DAVID 6.8 database (https://david.ncifcrf.gov/). The enrichment analysis results revealed that the 11 targets were enriched in the hematopoietic cell lineage, pathways in cancer, and microRNAs in cancer pathways. In addition, they were significantly enriched in the PI3K‐Akt signaling pathway, TNF signaling pathway, Ras signaling pathway and cAMP signaling pathway. These pathways are closely related to cell growth, metabolism, differentiation, proliferation, canceration, and apoptosis (Kauke et al., 2018; S. M. Kim et al., 2016; Li et al., 2018; Xiao & Kan, 2017). Interestingly, HTLV‐I infection was enriched, an infection that can cause T‐lymphocytic leukemia (Malik & Taylor, 2019).

**Table 1:** The major targets in identified targets of CML

ID	Gene	Subgragh	Eigenvector	Information	LAC	Betweenness	Closeness	Network
1	CREB1	2.55E+10	0.17698124	4.909849	12.11111	6047.7256	0.07061029	30.96654
2	FOS	2.54E+10	0.17668515	4.9251823	11.82143	5054.272	0.070361145	31.631813
3	KIT	2.14E+10	0.16219562	4.8032837	13.02326	3252.798	0.070404984	23.766201
4	BRCA1	1.87E+10	0.15158133	4.953085	12.43333	8358.199	0.070683904	37.659225
5	BCL2L1	1.86E+10	0.15115035	4.672883	14.58824	1093.766	0.06998348	20.309414
6	BCL6	1.37E+10	0.12977953	4.690172	10.28571	3923.2334	0.070070274	15.842359
7	CD44	1.21E+10	0.12183415	4.635592	10.25	3322.6553	0.06994017	15.123996
8	AKT2	9.70E+09	0.10923044	4.54799	10.07143	821.05743	0.06960986	15.814177
9	FLT3	9.20E+09	0.10639389	4.5717506	7.931035	1382.9755	0.06953846	12.190142
10	CHEK1	7.38E+09	0.09524421	4.77858	10.82927	2227.7275	0.069695726	25.106184
11	MCL1	7.05E+09	0.0931001	4.4049177	8.347826	1535.9803	0.069481455	11.391414

Abbreviation: CML, chronic myelogenous leukemia

**Figure 7:**
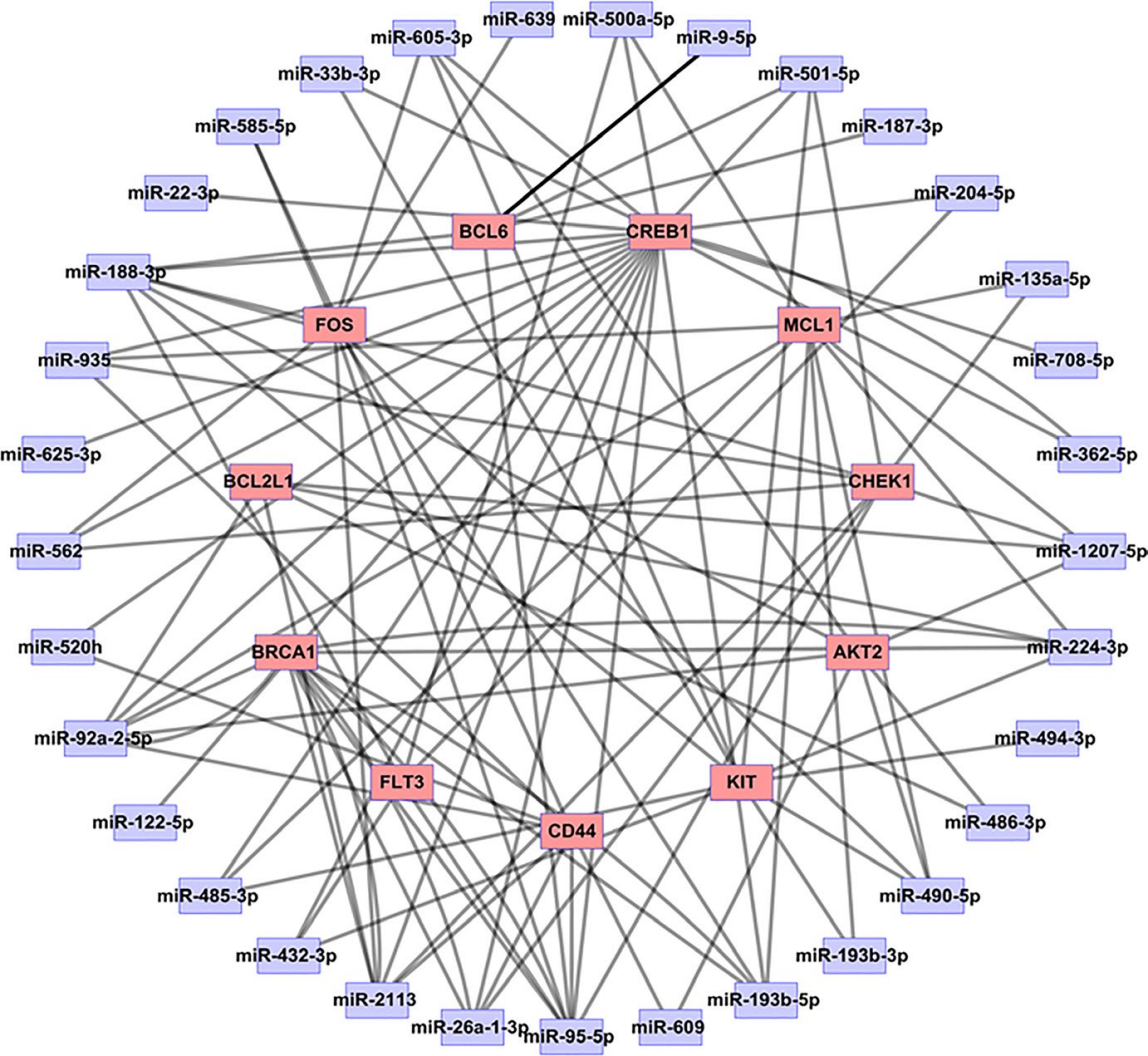
The microRNA‐Target network between 33 differentially expressed microRNAs and 11 major nodes. The network contains 44 nodes and 102 edges

We employed Cytoscape to construct microRNA‐Target network based on the relationship between differentially expressed microRNAs and 11 major nodes (Figure 7). The network display contains 44 nodes and 102 edges. There are 33 microRNAs that can predict 11 targets, including miR‐1207‐5p, miR‐362‐5p, miR‐708‐5p, miR‐204‐5p, miR‐501‐5p, miR‐500a‐5p, miR‐605‐3p, miR‐33b‐3p, miR‐22‐3p, miR‐188‐3p, miR‐935, miR‐625‐3p, miR‐562, miR‐520h, miR‐92a‐2‐5p, miR‐122‐5p, miR‐485‐3p, miR‐432‐3p, miR‐2113, miR‐26a‐1‐3p, miR‐95‐5p, miR‐193b‐5p, miR‐193b‐3p, miR‐490‐5p, miR‐494‐3p, miR‐224‐3p, miR‐135a‐5p, miR‐639, miR‐585‐5p, miR‐609, miR‐486‐3p, miR‐187‐3p, miR‐9‐5p.

## 4 DISCUSSION

Chronic myelogenous leukemia is a HSC‐driven hematological malignancy that is characterized by HSC translocation, leading to the expression of the active tyrosine kinase BCR/ABL. TKIs are very effective at inducing remission, but they fail to achieve therapeutic effects in the targeted treatment of LSCs. Leukemia stem cells maintain minimal residual tumors and can cause CML recurrence after discontinuing TKI treatment (Cheloni et al., 2017). Therefore, finding candidate targets for LSCs is of primary importance for the eradication of CML. With the rapid development of bioinformatics technologies, such as microarrays, research on disease mechanisms has reached the microRNA level. Therefore, in the present study, gene microarray expression profile data were used to identify key candidate microRNAs and DEGs in LSCs as new therapeutic targets and biomarkers to decipher the key signaling pathways that they are involved in.

Using bioinformatics analysis, we obtained 51 differentially expressed microRNAs from GSE90773 data expression profiles, including 26 up‐regulated and 25 down‐regulated differentially expressed microRNAs. The targets for differentially expressed microRNAs were predicted, and GSE11889 and GSE11675 were used. DEGs from the expression profiling analysis identified the predicted targets. Finally, after identification, a total of 116 targets were obtained, including 79 up‐regulated and 37 down‐regulated targets (GSE90773, GSE11889, and GSE11675 are all bone marrow LSCs derived from CML).

Chronic myelogenous leukemia is driven by BCR‐ABL1 expression and the subversion of normal signaling pathways (Koschmieder & Vetrie, 2018). We performed GO analysis and KEGG pathway enrichment analysis on the 116 genes to identify the key pathways for LSCs in CML. Recently, studies have found that BCR/ABL was involved in many signaling pathways, including the PI3K/AKT, Ras and JAK/STAT signaling pathways, which are activated by BCR/ABL, resulting in malignant proliferation of CML cells. These pathways are also involved in the resistance to apoptosis in CML cells and are associated with imatinib resistance in CML patients (Danisz & Blasiak, 2013). Pathway enrichment involves cytokine‐cytokine receptor pathways, including tyrosine kinase receptor activity and phosphokinase receptor and androgen receptor pathway activation; TKIs are currently the primary treatment for CML patients (Arrigoni et al., 2018). Notch signaling is critical for HSC self‐renewal and survival. A study found that inhibition of Notch led to hyperactivation of BCR‐ABL. The combined inhibition of Notch and BCR‐ABL may occur through the targeting of resting leukemia stem cells and differentiating leukemia cells (Aljedai, Buckle, Hiwarkar, & Syed, 2015). In addition, we also enriched the autophagic signaling pathway in HSCs from CML. Studies have shown that autophagy is involved in the regulation of LSC differentiation and is also closely related to the chemosensitivity of CML (Repsold, Pool, Karodia, Tintinger, & Joubert, 2017). Investigators also studied Realgar. The effect of transforming solution (RTS) on CML (K562) shows that RTS can induce autophagy with CML cells at a lower arsenic level by up‐regulating *LC3*, p62/SQSTM1 and mTOR inhibition, suggesting that autophagy can be a novel measure for CML therapy (Wang et al., 2018). We also enriched these pathways in the present study, demonstrating the accuracy of our research. Interestingly, we found that LSCs negatively regulated the extrinsic apoptotic signaling pathway via death domain receptors, which may be closely related to the apoptotic resistance of LSCs and can be used as a key candidate for reversing HSC resistance.

In our study, we obtained a total of 33 meaningful differentially expressed microRNAs of CML. Among these microRNAs, some have been confirmed to play a role in the pathogenesis of CML. MiR‐362‐5p is up‐regulated in fresh blood samples of CML cell lines and CML patients and is associated with growth arrest and DNA damage induced (GADD)‐45α down‐regulation and therefore can be used as a downregulator of GADD45α oncomiR, activating JNK1/2 and P38 signal transduction (Yang et al., 2015). Down‐regulation of miR‐494‐3p reduced TKI‐induced apoptosis, which indicated that miR‐494‐3p down‐regulation might contribute to the intrinsic TKI resistance of LSCs, and the result supports the development of novel therapies that target the regulation of miR‐494‐3p or its target to effectively eradicate LSCs (Salati et al., 2017). Overexpression and knockdown experiments of miR‐486‐3p demonstrated that miR‐486‐3p supported erythropoiesis while inhibiting megakaryocytopoiesis. miR‐486‐3p also favored granulocyte differentiation while inhibiting macrophage differentiation, thereby affecting the HPC genealogy(Bianchi et al., 2015). Researchers found that miRNA‐708 was highly expressed in TEL‐AML1, BCR‐ABL, E2A‐PBX1 and hyperdiploid (Yeh, Moles, & Nicot, 2016). In addition, we found that other microRNAs were involved in the regulation of tumor growth, proliferation, and apoptosis. For example, miR‐204‐5p inhibited the proliferation of hepatocellular carcinoma by directly regulating *SIX1* and its downstream factors (Chu et al., 2018). MiR‐500a‐5p was identified as an oxidative stress miRNA whose activity may define breast cancer progression and survival (Degli Esposti et al., 2017). MiR‐33b‐3p regulated cisplatin sensitivity in cancer cells, possibly by impairing the DNA damage response (Xu et al., 2016). MiR‐122‐5p inhibited the migration and invasion of gastric cancer cells by inhibiting *DUSP4* (Xu et al., 2018). However, the mechanism of action of these microRNAs in CML requires further exploration and experimental validation.

Through identification, we screened 11 CML HSC‐treated biomarkers and candidate targets, including *KIT*, *MCL1*, *FOS*, *BRCA1*, *FLT3*, *CD44*, *AKT2*, *CHEK1*, *BCL6*, and *BCL2L1*, and *CREB1*. *KIT* is a type III receptor tyrosine kinase that promotes cell survival and cells by activating downstream signaling pathways, proliferation and inhibition of apoptosis. *KIT* is a target for the treatment of CML (Kaitsiotou et al., 2017). *MCL1* is an antiapoptotic protein, and decreasing *MCL1* expression enhances IM‐induced apoptosis in CML cells (Shao et al., 2014). In this study, *MCL1* was significantly enriched in antiapoptosis pathways, especially in the resistance to exogenous withering upon the death signal, indicating that *MCL1* may be closely related to CML HSC resistance and apoptotic escape and may be used as a candidate target for the treatment of CML resistance. Genetic deletion of *FOS* inhibited tumor growth in a BCR‐ABL fusion protein kinase‐induced CML mouse model, and therefore, inhibition of high *FOS* expression reduced the intrinsic resistance of CML to TKI therapy (Kesarwani et al., 2017). The *BRCA1* is down‐regulated in CML patients, promoting abnormal mitosis and aneuploidy as well as altered DNA damage responses; studies have shown that BCR‐ABL strongly downregulates the *BRCA1* protein levels (Wolanin et al., 2010). Induction of *FLT3* in CML cells attenuates imatinib‐induced apoptosis, and *FLT3* is associated with disease progression and prognosis (Kim et al., 2010). Proliferation of K562 cells depends, to a large extent, on *CD44*, and down‐regulation of *CD44* lead to K562 cell G0/G1 arrest, whereby proliferation of the cell cycle is reduced. Therefore, *CD44* blockade may be beneficial for the treatment of CML (Chang et al., 2013). *AKT2* is a functional target of miR‐2278. Up‐regulation of miR‐2278 expression leads to inhibition of the proliferation of resistant leukemia cells and induction of apoptosis. *AKT2* inhibits leukemia cell proliferation and induces apoptosis upon experimental verification (Kaymaz et al., 2015). The *BCL6* proto‐oncogene is a key effector of FoxO in self‐renewing signaling in CML‐initiating cells and inhibits Arf and p53 in CML cells. The *BCL6* proto‐oncogene is required for colony formation and leukemic initiation. Studies suggest that pharmacological inhibition of *BCL6* may be possible, representing a new strategy to eradicate leukemia initiation cells in CML (Hurtz et al., 2011). *BCL2L1* is the gene for the primary antiapoptotic survivin protein. The study of *BCL2L1* expression by molecular changes strongly supports its involvement in ABT‐263/PP242‐induced CML‐BC progenitor cells. Apoptosis may also be a novel therapeutic target in CML (Lucas et al., 2016). CML can be treated using a combination of these 11 genes. For example, studies have shown that the combination of TKIs with *BCL6* and *MCL1* inhibitors may lead to the complete eradication of CML stem cells (Madapura et al., 2017).

There are some limitations in our research. Bioinformatics analysis based on chip samples only stays at the level of prebasic data analysis and prediction. The results are often determined by the original samples, the complexity of the original samples and the amount of samples can play an important role in interfering the results. Nonetheless, our research is based on real‐world sample data, and the results provide initial hypothesis for in vivo and in vivo target validation studies, which can effectively reduce the shortcomings of previous studies with low innovation and high clinical loss.

## 5 CONCLUSION

In this study, we used multiple microarray datasets and performed bioinformatics analysis. During the analysis, a total of 51 microRNAs and 116 target genes for differentially expressed microRNAs were identified. Through the construction of protein‐protein interaction networks, module analysis and topology analysis of networks were performed. Finally, we obtained a total of 33 differentially expressed microRNAs and 11 key targets. The key targets were mainly enriched in the PI3K/AKT, Ras, JAK/STAT, FoxO and Notch signaling pathways and were resistant to endogenous and exogenous apoptosis. These findings can significantly improve our understanding of the molecular pathogenesis of LSCs. These key candidate genes and pathways may encompass the thorough treatment of CML strategies.

## CONFLICT OF INTERESTS

There are no conflicts of interest to declare.

## AUTHOR CONTRIBUTIONS

Conceptualization, C.G.S., H.Y.L., L.J.L.; Methodology, C.G.S., H.Y.L., L.J.L., J.Z., C.L., C.Z., J.Y., C.D.G., G.X.L.; Software, H.Y.L., L.J.L., J.Z., C.L., C.Z.; Validation, C.L., C.Z., J.Y., C.D.G., G.X.L.; Visualization, H.Y.L., L.J.L., J.Z., C.L.; Roles/Writing—original draft, H.Y.L., L.J.L.; Writing—review & editing, C.G.S., H.Y.L., L.J.L., J. Z.
